# Case report: Liver PEComa after kidney transplantation in recipient with tuberous sclerosis complex

**DOI:** 10.3389/fonc.2024.1386569

**Published:** 2024-07-04

**Authors:** Marcin Dymkowski, Paulina Kalman, Piotr Niecikowski, Łukasz Koperski, Maciej Kosieradzki

**Affiliations:** ^1^ Department of General and Transplantation Surgery, Medical University of Warsaw, Warsaw, Poland; ^2^ Department of Pathology, Medical University of Warsaw, Warsaw, Poland

**Keywords:** PEComa, epithelioid angiomyolipoma, organ transplantation, kidney transplant, tuberous sclerosis

## Abstract

**Introduction:**

Perivascular epithelioid cell tumors (PEComa) are rare tumors of mesenchymal origin that exhibit perivascular epithelioid cell phenotype. One of its most common localizations is uterus, whereas only a few studies reported PEComa localization as liver. There is a correlation between the presence of PEComa and tuberous sclerosis complex (TSC). TSC is a rare disease which leads to the development of mostly non-cancerous tumors in various organs. We would like to present a case of a kidney transplant recipient with a PEComa detected post-transplant in the liver.

**Case report:**

A 27-year-old patient, 3 years after kidney transplantation (KTx) due to chronic renal failure in the course of autosomal dominant polycystic kidney disease and concomitant TSC, was admitted to the Clinic and Department of General and Transplant Surgery for abnormal findings in computed tomography (CT). A CT scan was conducted for oncological follow-up after a kidney transplant (KTx) because before the transplantation, a small cystic lesion measuring 7 mm in diameter was removed from the donor kidney and diagnosed as papillary renal cell carcinoma (PRCC). Two tumors in the liver were detected - one 27mm in diameter in segment VII/VIII and the other 8mm in diameter in segment II/III. Because of typical radiological signs hepatocellular carcinoma was suspected, but the serum level of alpha fetoprotein was within normal limits and liver function was preserved. The intraoperative biopsy and the radiofrequency ablation (RFA) of the larger tumor were performed three months later. In the histopathological examination benign PEComa (HMB45 +, Melan A +) was detected.

**Conclusion:**

The oncological surveillance made it possible to detect liver lesion in early stage and in 3,5-year follow-up no sign of recurrence of PEComa was found. This case is the second to show RFA as treatment method of liver PEComa and first in kidney transplant recipient.

## 1 Introduction

Perivascular epithelioid cell tumors (PEComa) are a differentiated family of lesions originating from mesenchymal tissue localized in many sites. In 1992, Bonetti first introduced the notion of PECs to describe a “unusual atypical cell type” with a perivascular distribution and dual melanocytic and myoid development (1). The concept of PEComa as a group was introduced by Pea et al. (2) in 1996. In the 5th edition of WHO Soft Tissue and Bone Tumors (2020) classification PEComa is described as a group composed of angiomyolipoma (AML), lymphangioleiomyomatosis (LAM) and epithelioid angiomyolipoma (eAML) which is a synonym of PEComa (3). In the epidemiology of PEComas, there is a notable predominance of female patients and a relatively young median age of 43.5 years. While the kidneys, uterus, and gastrointestinal tract are the most frequently affected areas, the diversity in primary tumor locations indicates that these tumors can originate in any organ (4). PEComa of the liver are extremely rare and as difficult to evaluate as all types: classic AML (cAML) and epithelioid AML (eAML) (5). Moreover, liver PEComa may be similar and challenging to distinguish from other liver lesions including hepatocellular carcinoma (HCC), adenoma, focal nodular hyperplasia (FNH), lipoma, myelolipoma, other primary hepatic tumors and even metastatic lesions. The rarity of hepatic PEComa leads to preoperative misdiagnosis of the lesion in many cases, hence the diagnostic and therapeutic strategies are not established yet. Surgical resection remains the primary treatment for hepatic PEComa (6). mTOR inhibitors (sirolimus/everolimus) and antracyklin-based and gemcitabine-based chemotherapy were most often prescribed (4). Here we present a case of a kidney transplant recipient with tuberous sclerosis complex (TSC) with liver PEComa which was misdiagnosed and treated with radiofrequency ablation (RFA). To the best of our knowledge, it is the second case of liver PEComa treated with RFA (7) and the first in a kidney transplant recipient.

## 2 Case report

The patient was a male kidney transplant recipient, due to end-stage chronic kidney disease in course of autosomal dominant polycystic kidney disease (ADPKD). Moreover, the patient was diagnosed with TSC, secondary hypertension and nicotinism.

At the age of 24, pre-emptive kidney transplantation (KTx) was performed with satisfactory graft function. The immunosuppressive treatment was standard for KTx - prednisone, mycophenolate mofetil and tacrolimus. During back-table preparation of a graft, a cystic lesion, 7 mm in diameter, was excised and sent for routine histopathological examination, which revealed papillary renal cell carcinoma (PRCC) type I (G1, pT1a, F0). The patient was put on an oncological surveillance protocol. Then, in 2.5-year follow-up no lesions were found.

**Figure 1:**
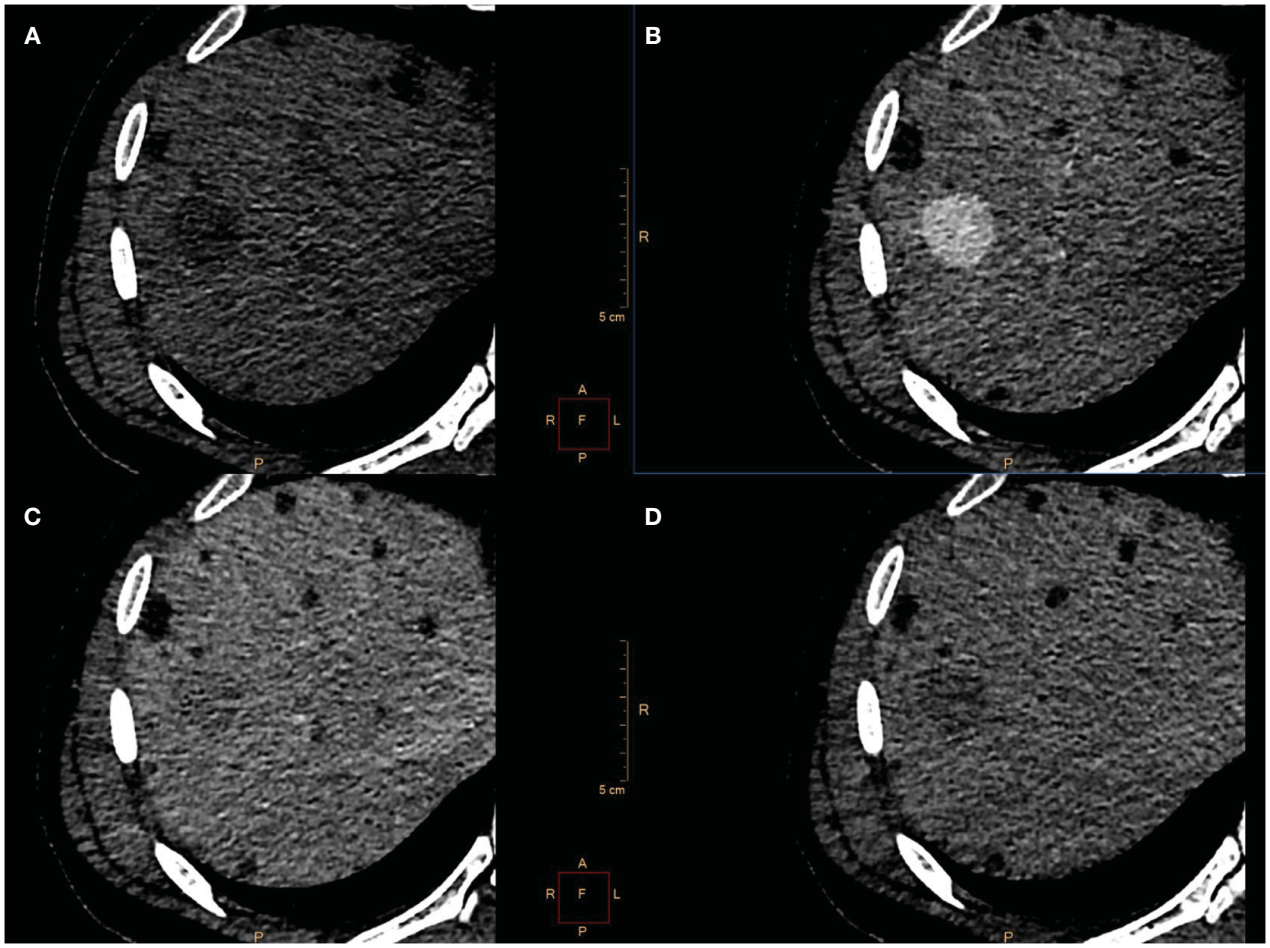
CT scan showed a 27 mm-large tumor localized in segment 7/8 **(A)** with rapid contrast enhancement in the arterial phase **(B)** and washout in venous **(C)** and delayed phase **(D)**. Because of radiological characteristic HCC was suspected.

3-years after KTx, the patient was admitted for abnormal findings on abdominal computed tomography scan (CT) which revealed two liver lesions. 27 mm in diameter in segments 7/8 and 8 mm in segments 2/3. Both showed similar CT pattern with rapid enhancement in the arterial phase and washout in the venous phase (**Figure 1**). The patient didn’t report any symptoms.

CT scans were reassessed in our center. The smaller lesion was considered to be a group of small vessels that mimicked the enhancement pattern corresponding to HCC. Considering larger lesion, radiological findings suggested HCC, but serum alpha-fetoprotein was within normal limits. Metastasis of PRCC was also taken into consideration as a differential diagnosis. Taking into account preoperative diagnosis of HCC based on CT and small size of tumor (<3cm) the patient was scheduled for radiofrequency ablation (RFA) of a larger tumor with intraoperative fine needle biopsy to confirm the diagnosis. Open surgery was performed with wide right subcostal incision. Intraoperative USG identified the tumor (25x30 mm) in segment 6/7 and a core needle biopsy was taken under USG guidance. Two RFA needles (30 mm and 40 mm) were positioned and RFA was performed (45 min, E=110 kJ, 225/250 W).

**Figure 2:**
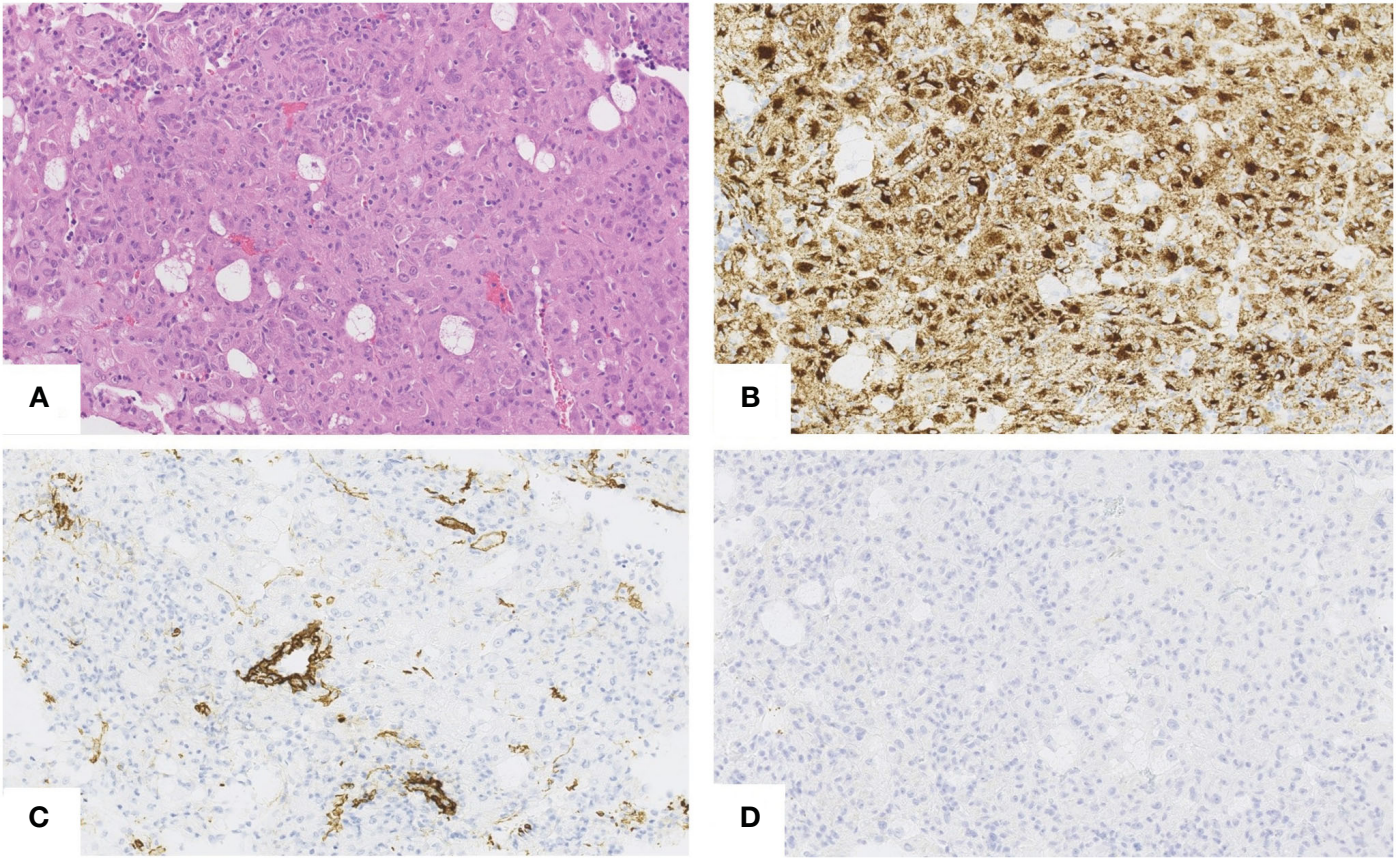
Hepatic PEComa. Hematoxylin andeosin (H&E) staining revealed atypical epithelioid cells with abundant eosinophilic cytoplasm. No necrosis and no mitosis have been noticed **(A)**. Immunohistochemically the tumor cells were diffusely positive for HMB45 **(B)** and focally positive for SMA **(C)** Negative staining for HepPar1 **(D)** enable to exclude the hepatocyte origin of tumor. [**(A–D)**,scale bars 50µm.]

**Figure 3:**
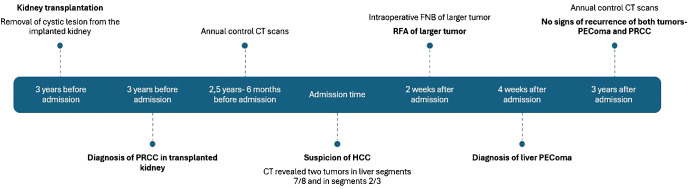
Time line of case history. PRCC, papillary renal cell carcinoma. CT, computed tomography. HCC, hepatocellular carcinoma. FNB, fine needle biopsy. RFA, radiofrequency ablation.

Microscopically, the tumor cells were composed of epithelioid cells with abundant eosinophilic cytoplasm (**Figure 2A**). The tumor cells showed cellular and nuclear pleomorphism with prominent nucleoli. Lipid component of some cells was observed. The nests or groups of neoplastic cells were surrounded by thin-walled capillary vessels. No mitosis or neoplastic necrosis has been observed. Additional immunohistochemical staining results showed that the tumor cells were diffusely positive for HMB45 (**Figure 2B**), focally for Melan-A and SMA (**Figure 2C**) and negative for HepPar-1 (**Figure 2D**), S100, AFP, keratin AE1/AE3, Glypican3, CD117 and DOG-1 markers. Perivascular epithelioid cell tumor was diagnosed. As PEComa is unusual in the liver the metastatic disease was suspected, but in terms of no findings in radiological examination suggesting primary origin in other site, primary hepatic PEComa was diagnosed. In follow-up CT scans, the gradually shrinking ablation zone was visible and there were no signs of recurrence of neither PEComa (3-year follow-up), nor PRCC (6-year follow-up). The timeline with relevant data from the episode of care is displayed in **Figure 3**.

## 3 Discussion

The histogenesis and pathophysiology of PECs remain unknown. One idea is that it stems from undifferentiated neural crest cells that can express melanocyte and smooth muscle phenotypes. Other possible causes include smooth muscle and pericytic origins (8). Genetic studies indicate that PEComa can be sporadic or a component of TSC (including TSC1 and TSC2), implying that it is a TSC-associated tumor (9) which is an autosomal dominant genetic condition produced by germline mutations in SC1 on chromosome 9 or TSC2 on chromosome 16 (10). Among soft tissue PEComas, fibroma-like PEComas are strongly related with this syndrome (11). Although most soft tissue PEComas are not related with TSC, many exhibit TSC2 changes. TFE3 rearrangements represent an additional molecular route for carcinogenesis. TFE3-rearranged PEComas, with few exceptions, are not seen in TSC patients, are more common in young people, and are typically found in the gynecologic, genitourinary, and gastrointestinal tracts, as well as soft tissue sites (12). In the literature, various TFE3 partners have been described and continue to be described. The presence of the TFE3 fusion protein most likely replaces MiTF in these PEComas, explaining the absence of MiTF expression and the reduced expression of Melan A. TFE3 rearrangements and TSC1/2 changes were previously thought to be mutually incompatible, but a recent report of a TCS1-mutated PEComa with a TFE3-altered phenotype calls this conclusion into question (13). As a result, performing molecular biology studies in PEComas is critical for both understanding tumor development and selecting the most appropriate treatment (14).

In recent years many classifications of mesenchymal tumors described tumors de-rived from PECs. In 2004 WHO (5) divided AML into classic AML (cAML) and epithelioid AML (eAML). We point out the variety of phenotype expressions of AML-like tumors. There are no strict criteria for fatty, muscular and vascular content proportion characteristic to a subgroup. Despite accepted classifications of PEComa, there is substantial confusion in the use of nomenclature associated with this group of tumors. Terms such as epithelioid AML, hepatic epithelioid AML, hepatic PEComa, PEComa, hepatic AML (HAML) and monomorphic epithelioid AML can be found. Unrecommended terminology is still in use. eAML is often included in the analysis with cAML which is confusing (15, 16) and makes it impossible to distinguish between the characteristics of both groups. HAML, first described by Ishak et al. (17) in 1976, is now also classified as PEComa. Recent papers reported a prevalence of hepatic PEComa to be 300-600 cases, but it is problematic to estimate true epidemiology (18, 19). There is a higher prevalence of various neoplasms in organ transplant recipients (6). Therefore, there is no available data regarding solid tumor transplantation and PEComa occurrence.

The association between TSC and renal AML, first described in 1911 (20), is observed in 50% of cases, while the association between TSC and liver PEComa is only observed in 5% to 15% of cases (19, 21). In TSC patients, liver PEComa is frequently accompanied with renal AML. A recent retrospective analysis found that among 25 patients with liver PEComa, 88% also had renal AML, and TSC2 patients had a higher prevalence of liver PEComa than TSC1 patients (18% vs 5%; P = 0.037) (22, 23).

The majority of TSC patients (more than 80%) acquire some sort of renal dysfunction during their lives (24). AML and cystic kidney disease are two of the most prevalent renal symptoms of TSC, with ADPKD accounting for only about 2% of patients (25). The characteristic ADPKD renal phenotype may arise in the context of TSC disease due to substantial deletions of the PKD1 and TSC2 genes on chromosome 16p13. This disorder, also known as TSC2/ADPKD1 contiguous gene syndrome, is diagnosed when kidney abnormalities indicative of ADPKD phenotype coexist with TSC phenotype (26, 27).

Preoperative diagnosis of hepatic PEComa is difficult to make (15, 28) and is often an incidental finding (29). Other hepatic lesions, such as FNH, HCC, hemangioma, adenoma and others are more probable and hence are always put forth in diagnostic process. In our case, HCC and PRCC metastasis were considered differential diagnoses. On CT, PEComa shows similar characteristics to HCC with rapid enhancement in the arterial phase and washout in the venous phase. AFP was within normal limits in serum and negative in staining. Sonazoid-enhanced ultrasonography is reported to be helpful in the diagnosis of hepatic PEComa (30–32).

PEComas show a wide range of imaging results. Hepatic PEComas can be of any echogenicity and have blood flow within or around the lesion. AML can be easily diagnosed in cases where fat covers more than 50% of the tumor. Diagnosis is difficult in tumors with no or little fat. Non-enhanced CT and MRI scans indicate tumors with low attenuation, low signal intensity on T1, and high signal intensity on fat-suppressed T2 and DWI images. Most liver tumors have nonspecific signs. Previous research have identified hypervascularity and arteriovenous connections as characteristics of PEComa (33, 34). Nie et al. discovered a varied enhancing scheme. Enhancements in the arterial phase were observed with fast washout (n = 9), delayed washout (n = 7), and persistent washout. The study found enhancement in the late phases (n = 4) and an undefined heterogeneous enhancement pattern (n = 2). The radiological variance in tumors can be attributed to the presence of several components, including adipose tissue, blood vessels, and smooth muscle cells, which can range from less than 10% to over 90% (35).

HMB45, SMA and focally Melan-A were positive, while HepPar-1, S100, AFP, keratin AE1/AE3, Glypican3, CD117 and DOG-1 markers were negative. Those findings are comparable with other authors (15, 36–38). Immunohistochemical staining is the basis in differentiation from other liver tumors and metastases. A core-needle biopsy can allow a preoperative diagnosis but is not perfect in all cases (39).

PEComa may exhibit both benign and malignant characters, however, the benign one is more common. Repetitively some criteria for malignancy are included in papers by Folpe et al. who proposed classification into 3 groups: “benign”, “uncertain malignant potential” and “malignant”. Criteria of malignancy in this classification were: tumor size >5 cm, nuclear pleomorphism, high nuclear grade or cellularity, mitotic rate ≥1/50 HPF, infiltrative growth, necrosis and vascular invasion (40). In liver PEComa, those criteria were not validated. Case reports of hepatic lesions with malignant behavior were reported and most of them did not meet the criteria (18, 28, 41–44).

Treatment of hepatic PEComa in nearly all cases is surgical resection including tumorectomy, segmentectomy and lobar resection (16, 19). In one case of recurrent malignant PEComa a liver transplantation was performed (45). Guan et al. (7) reported a combined treatment of transarterial embolization followed by RFA in a patient with hepatic PEComa. RFA is usually a palliative treatment but can be curative in small tumors in the early stage. RFA has no significant difference in long-term survival compared to surgical resection and liver transplantation in treatment of HCC. It also offers benefits such as lower risk of complications, lower cost, normal tissue preservation, and shorter hospital stay. RFA is the third local curative method for HCC, with tyrosine kinase inhibitors, immune checkpoint inhibitors, and transarterial chemoembolization being palliative treatments.

In our case treatment with RFA was used as the tumor size was <3cm and radiological examination suggested HCC. Preoperative tumor biopsy is indicated in case of uncertain radiological diagnostic (46). Use of RFA in early-stage HCC was shown inferior in overall survival and disease-free survival in comparison to liver resection. However, in tumors smaller than 3cm outcomes were comparable with lower rate of complications (47). In terms of liver PEComa there is lack of data supporting or contradicting this kind of treatment. In our case, the patient is disease free after a 3.5-year follow-up. Chemo- and radiotherapy have not been widely investigated for PEComa. PEComas are caused by the proliferation of PECs with mutations that result in loss of TSC gene activity and overexpression of mTOR kinase. Inhibitors of mTOR may offer new therapy options. However, sirolimus was successfully used in some cases (36) and was utilized as a systemic neoadjuvant therapy to reduce tumors and enable surgical removal. Based on prior understanding of mTOR pathway activity in other primary malignancies, the inclusion of hormone treatment was tested in a small case series of progressive PEComas following mTOR inhibitors and revealed an intriguing effectiveness signal. Recently, two molecular subgroups of PEComas were proposed: type 1, which responds to mTOR inhibitors, and type 2, which responds to c-MET inhibitors (48). c-MET inhibitors may be more effective in TFE3-altered PEComas because TFE3 fusions increase MET signaling through transcriptional up-regulation (49). Among systemic medicines, mTOR inhibitors are by far the most commonly utilized across the board, either as a curative treatment in conjunction with a radical treatment (surgery or radiotherapy) or in a palliative situation. However, as of now, there is no clear advice, and it has to be seen if it can or cannot be called the gold standard for all patients or simply for a subset of patients, such as TSC2-mutated patients. In an attempt to anticipate the response to mTOR inhibitors, an intriguing study found that the degree of phosphorylated S6 ribosomal protein expression, which indicates mTOR pathway activation, was predictive of early tumor response to the treatment. However, no studies have been conducted to compare the response to mTOR inhibitors in PEComas with and without TFE3 translocation (4, 50).

Treatments for malignant PEComas included transarterial embolization, radiofrequency ablation, and stereotactic body radiation therapy (7, 51).

## 4 Conclusions

Little is known of PEComa, especially of liver PEComa and its epithelioid subtype. The rarity of liver PEComa makes it very difficult to diagnose preoperatively and weigh therapeutic decisions reasonably.

## Data availability statement

The original contributions presented in the study are included in the article/supplementary material. Further inquiries can be directed to the corresponding author.

## Ethics statement

Ethical approval was not required for the study involving humans in accordance with the local legislation and institutional requirements. Written informed consent to participate in this study was not required from the participants or the participants’ legal guardians/next of kin in accordance with the national legislation and the institutional requirements. Written informed consent was obtained from the individual(s) for the publication of any potentially identifiable images or data included in this article.

## Author contributions

MD: Conceptualization, Investigation, Project administration, Visualization, Writing – original draft, Writing – review & editing. PK: Project administration, Visualization, Writing – original draft, Writing – review & editing. PN: Conceptualization, Writing – review & editing. LK: Resources, Supervision, Writing – review & editing. MK: Conceptualization, Resources, Supervision, Writing – review & editing.
